# Prediction of orthognathic surgery plan from 3D cephalometric analysis via deep learning

**DOI:** 10.1186/s12903-023-02844-z

**Published:** 2023-03-18

**Authors:** Mengjia Cheng, Xu Zhang, Jun Wang, Yang Yang, Meng Li, Hanjiang Zhao, Jingyang Huang, Chenglong Zhang, Dahong Qian, Hongbo Yu

**Affiliations:** 1 Department of Oral and Cranio-Maxillofacial Surgery, Shanghai Ninth People’s Hospital, College of Stomatology, Shanghai Jiao Tong University School of Medicine, Shanghai, 200011 China; 2 National Center for Stomatology & National Clinical Research Center for Oral Diseases, Shanghai, 200011 China; 3 Shanghai Key Laboratory of Stomatology &, Shanghai Research Institute of Stomatology, Shanghai, 200011 China; 4 Mechanical College, Shanghai Dianji University, Shanghai, 201306 China; 5 School of Computer & Computing Science, Hangzhou City University, Hangzhou, 310000 China; 6 Shanghai Lanhui Medical Technology Co., Ltd, Shanghai, 200333 China; 7 School of Biomedical Engineering, Shanghai Jiao Tong University, Shanghai, 200030 China

**Keywords:** Dento-maxillofacial deformity, Orthognathic surgery, Virtual surgical planning, Deep learning, Regression prediction, Transformer

## Abstract

**Background:**

Preoperative planning of orthognathic surgery is indispensable for achieving ideal surgical outcome regarding the occlusion and jaws' position. However, orthognathic surgery planning is sophisticated and highly experience-dependent, which requires comprehensive consideration of facial morphology and occlusal function. This study aimed to investigate a robust and automatic method based on deep learning to predict reposition vectors of jawbones in orthognathic surgery plan.

**Methods:**

A regression neural network named VSP transformer was developed based on Transformer architecture. Firstly, 3D cephalometric analysis was employed to quantify skeletal-facial morphology as input features. Next, input features were weighted using pretrained results to minimize bias resulted from multicollinearity. Through encoder-decoder blocks, ten landmark-based reposition vectors of jawbones were predicted. Permutation importance (PI) method was used to calculate contributions of each feature to final prediction to reveal interpretability of the proposed model.

**Results:**

VSP transformer model was developed with 383 samples and clinically tested with 49 prospectively collected samples. Our proposed model outperformed other four classic regression models in prediction accuracy. Mean absolute errors (MAE) of prediction were 1.41 mm in validation set and 1.34 mm in clinical test set. The interpretability results of the model were highly consistent with clinical knowledge and experience.

**Conclusions:**

The developed model can predict reposition vectors of orthognathic surgery plan with high accuracy and good clinically practical-effectiveness. Moreover, the model was proved reliable because of its good interpretability.

**Supplementary Information:**

The online version contains supplementary material available at 10.1186/s12903-023-02844-z.

## Introduction

**Fig. 1:**
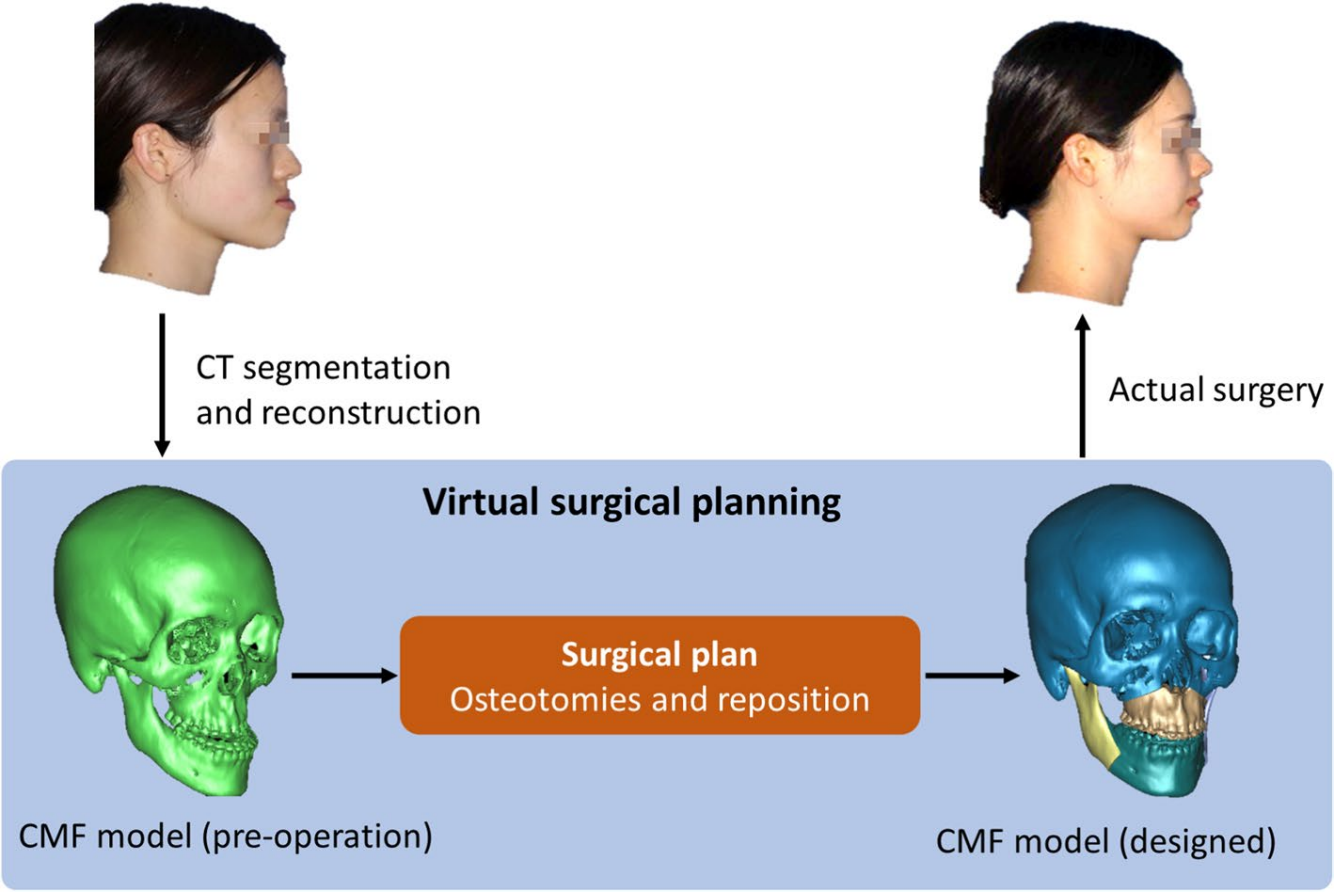
VSP procedures for orthognathic surgery. Before surgery, patient's computed tomography (CT) data were collected to reconstruct individual craniomaxillofacial (CMF) model. A patient-specific normal CMF structure can be designed through performing osteotomies on jawbones and repositioning bony segments. The determined jaws' position and occlusion can be transferred to actual surgery through surgical guides

Resulted from an abnormal growth of facial bones (mainly maxilla and mandible), patients with dento-maxillofacial deformities have facial malformations and dysfunction, such as abnormal or unaesthetic facial features, low-efficient mastication, temporomandibular joint (TMJ) diseases, airway obstruction and etc. Orthognathic surgeries are used to correct dento-maxillofacial deformity and rebuild functional occlusion through performing osteotomies on jawbones, repositioning bony segments and fixing them on new position [1, 2]. Virtual surgical planning (VSP) approaches enable surgeons to simulate surgical procedures on patient's virtual craniomaxillofacial (CMF) model and make a surgical plan. Then, the determined jaws' position and occlusion will be transferred to actual surgery through surgical guides (Fig. 1).

However, making an orthognathic surgery plan is sophisticated. Firstly, 3-dimensional morphological characteristics of face, jawbones and dentition need to be evaluated based on measurements of radiographic image data (i.e. cephalometric analysis) and clinical examination [3]. Then, surgery types, direction and amount of bony segments' movement are determined to accomplish an individualized, optimal surgical plan. Unfortunately, there are no specific and reliable formulas or fixed associations between quantified morphological characteristics and surgical plan. Therefore, making an orthognathic surgery plan depends mostly on surgeon's experience, esthetic sense and postoperative prediction ability.

Artificial intelligence (AI) provides an ideal alternative to cephalometry analysis and surgical planning. Some researchers attempted utilizing deep learning (DL) or other machine learning methods to predict a normal bone structure to assist orthognathic surgery planning. Wang et al. (2015) developed a surface deformation network to predict patient-specific normal jaw shape using sparse representation [4]. They constructed a normal subjects dictionary as reference to estimate and generate a patient-specific normal jaw shape. Because of low robustness in real-world data and the reliance of manual landmark digitization, the surface deformation network was improved by employing a geometric deep learning method named Pointnet +  +  [5]. Xiao et al. (2021) proposed SDNet (still require manual landmark digitization) in [6] and DefNet (end-to-end method, not require manual landmark digitization) in [7], both of which demonstrated impressive performance when validated by 24 real patient cases. The mean landmark distance between the estimated and ground-truth bony surfaces were 3.70 ± 0.72 mm for SDNet and 4.01 ± 0.85 mm for DefNet. In [8], Ma et al. proposed a method to predict postoperative 3D coordinates of 11 landmarks, which were transformed into vectors of jawbones' movement to assist VSP of orthognathic surgery. This method was tested on 6 real patients' data, and the average prediction accuracy was 5.4 ± 0.6 mm at the landmark level. Despite of their innovated work, the prediction accuracy of aforementioned studies cannot meet clinical standard (less than 2 mm). In terms of the methodology, they all neglected the impact of soft tissue features and patients' personal characteristics on orthognathic surgery plan. To improve prediction accuracy, more information of patients' characteristics should be considered as input features.

To tackle the limitations of previous researches and improve prediction accuracy, a regression neural network, namely VSP transformer, was proposed. There are three main contributions of this study: 1) Patients' CMF structural characteristics (including soft tissue profile) were quantified using 3D cephalometric analysis to assist the model in prediction accuracy. 2) the contribution of each input feature in the model was analyzed using permutation importance (PI) method to provide interpretable information for clinicians. 3) To the best of our knowledge, the dataset used in this study was the largest real patient dataset of dento-maxillofacial deformity with detailed baseline characteristics so far.

## Materials and methods

### Data collection

All datasets were collected from Department of Oral and Craniomaxillofacial Surgery, Shanghai Ninth People’s Hospital. This research was approved by the Research Ethics Committee in Shanghai Ninth People’s Hospital (IRB No. SH9H-2022-TK12-1). 383 samples in development dataset were retrospectively collected from patients who underwent treatment in 2019 and 2020. 49 samples in clinical test dataset were prospectively collected from patients in 2021 and 2022. These two datasets shared same inclusion and exclusion criteria. Inclusion criteria were 1) patient who was diagnosed with skeletal malocclusion and orthognathic surgery was required, 2) orthognathic surgery planning involved at least maxilla or mandible, and osteotomy methods were Le Fort I for maxilla, bilateral sagittal split ramus osteotomy (BSSRO) or/and genioplasty for mandible. The exclusion criteria were 1) congenital dentofacial deformities, 2) segmental osteotomies were used for maxilla or mandible.

### Quantify morphological characteristics using 3D cephalometric analysis

**Fig. 2:**
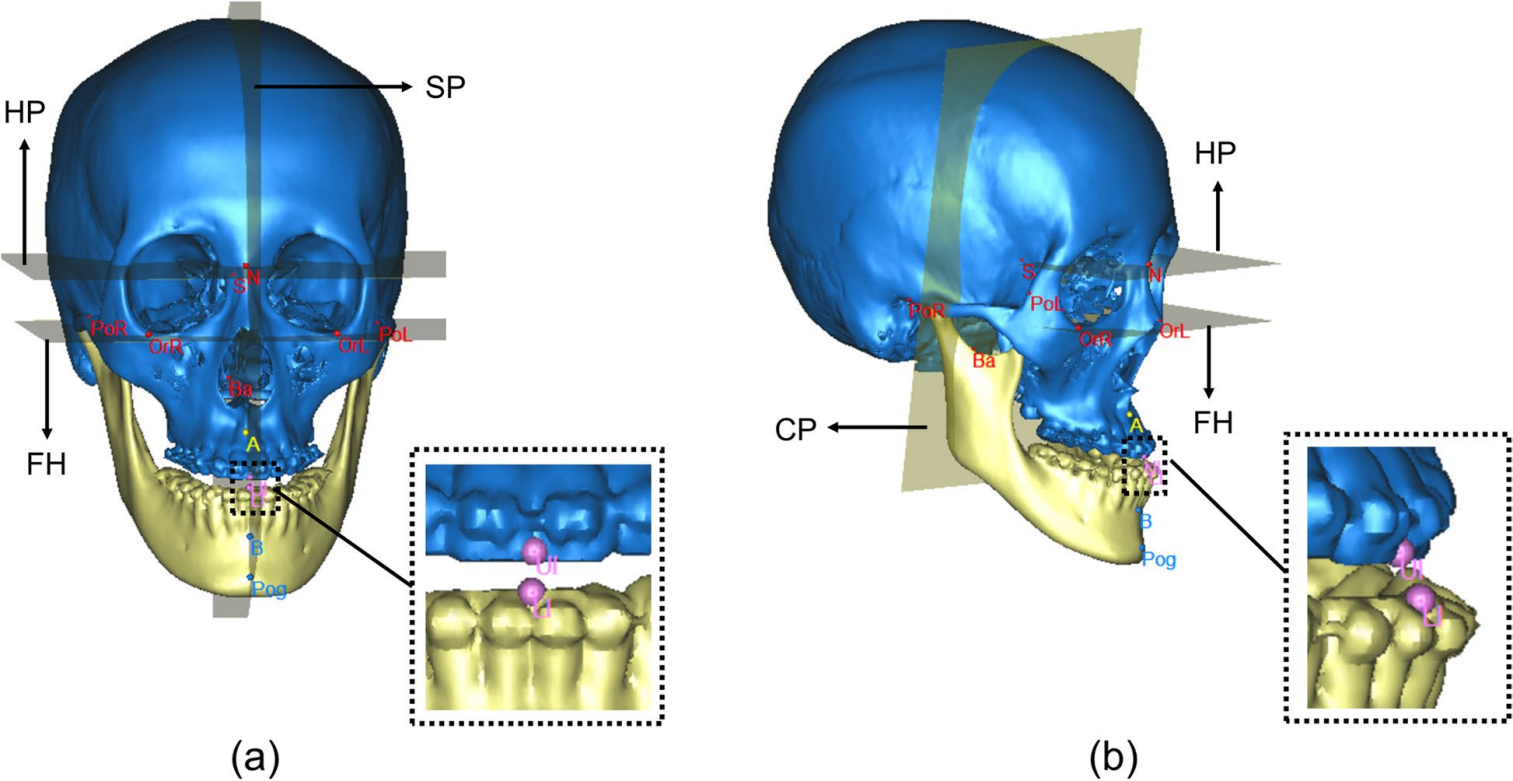
Four reference planes of CMF structure and five key landmarks. **a** frontal view. **b** lateral view. Frankfurt horizontal plane (FH) was defined as the plane passing through orbitale point of two sides (OrR, OrL) and midpoint of bilateral porion points (PoR, PoL). Horizontal plane (HP) was defined as the plane parallel to FH and through nasion (N). Sagittal plane (SP) was defined as the plane normal to FH and through N point and basion (Ba). Coronal plane (CP) was defined as the plane through sella point (S) and normal to SP and FH. Five key landmarks include A point (A), midpoint of U1 incisal tip (UI), midpoint of L1 incisal tip (LI), B point (B) and pogonion point (Pog)

Orthognathic surgery planning of all patients was conducted using Proplan CMF software (Materialise company, Belgium). All surgical plans were ultimately approved by chief surgeons and then precisely transferred into actual surgeries through individualized surgical guides. Therefore, the difference between preoperative CMF models and designed CMF models was considered as ground truth for training process of the deep learning model. A customized 3D cephalometric analysis template was designed and inserted into Proplan CMF software to quantify and standardize measurements in all samples. Required anatomic landmarks were digitized by 4 experienced CMF surgeons. In our designed cephalometric analysis, four reference planes, including Frankfurt horizontal plane (FH), horizontal plane (HP), middle sagittal plane (SP) and coronal plane (CP) were established to build 3D coordinate system (Fig. 2). It’s worth noting that SP was adjusted to being consistent with facial midline, and the latter was determined through clinical examination of patients in natural head position (NHP). Then, twelve key cephalometric variables were acquired to represent morphological characteristics of dentition, jawbones and facial soft tissue (detailed definitions were illustrated in Supplementary Table 1.) Moreover, surgery plan was quantified as landmark-based reposition vectors, namely sagittal and vertical movement of point A, UI, LI, B, and Pog (Fig. 2). These five landmarks were chosen because they are crucial for facial appearance changes and frequently referred in standard facial aesthetics assessment indexes proposed by experts [9, 10]. At last, twelve key cephalometric variables and gender were selected as input features. Reposition vectors of five key landmarks in two directions were set as output variables.

### Data preprocessing

Since input features have different magnitude ranges and units (degree or millimeter), their values were normalized between 0 and 1 according to Min–Max normalization method (Formula 1).

1 $${{\varvec{x}}}_{{\varvec{i}}{\varvec{j}}}^{*}=\frac{{{\varvec{x}}}_{{\varvec{i}}{\varvec{j}}}-{\varvec{min}}({{\varvec{x}}}_{{\varvec{i}}})}{{\varvec{max}}({{\varvec{x}}}_{{\varvec{i}}})-{\varvec{min}}({{\varvec{x}}}_{{\varvec{i}}})}$$

Where ${\chi }_{ij}$,${\chi }_{ij}$ represent all data and the value of each feature respectively. ${max(x}_{i})$ and ${min(x}_{i})$ are the maximum and minimum values in each input column of the training set. Clinical test set was also normalized by the threshold of the training set to reduce model derived prediction error.

**Fig. 3:**
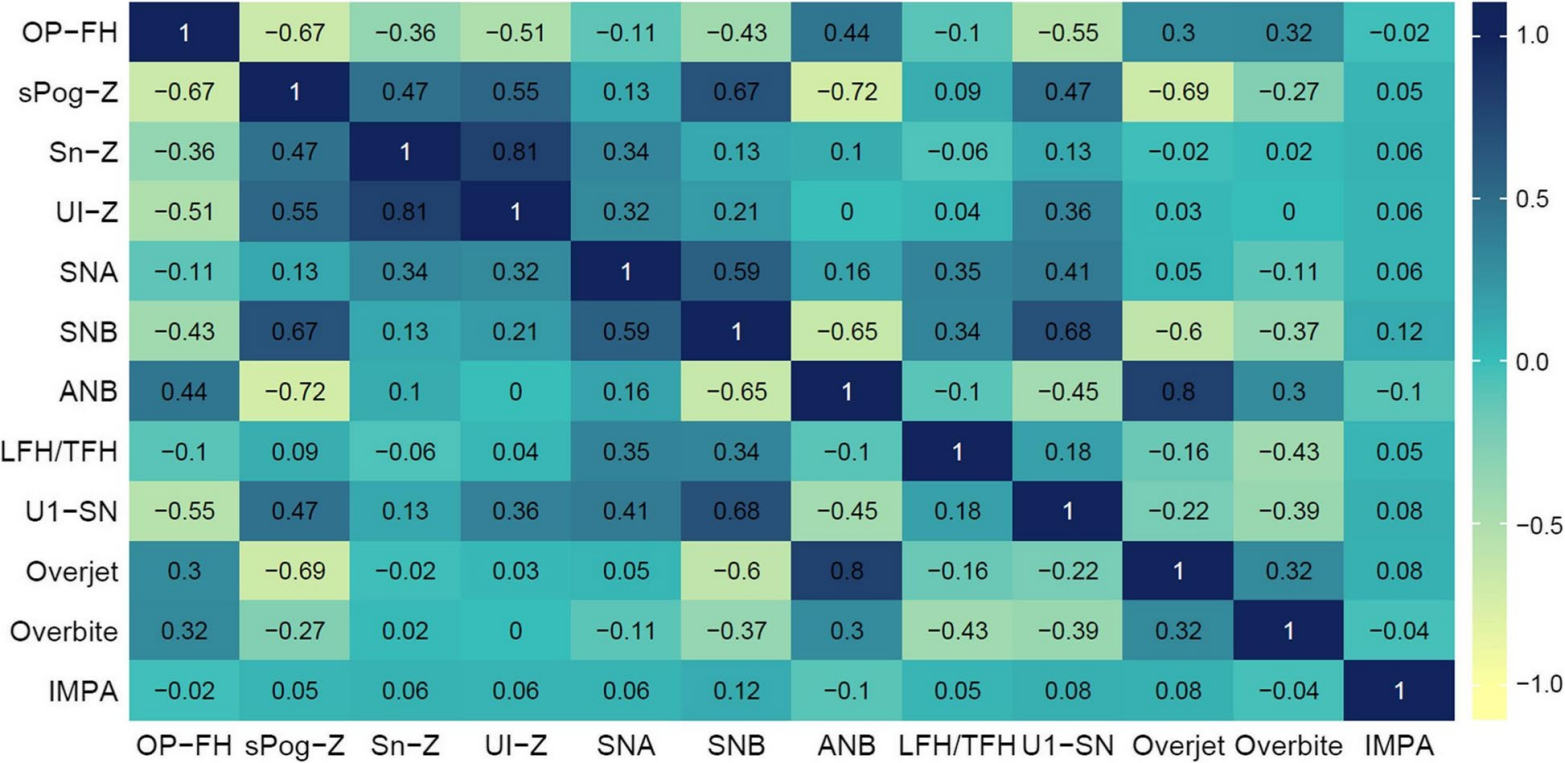
Correlation heatmap of input features. The values are Spearman correlation coefficients. Blue indicates positive correlations and yellow indicates negative correlations

Then, correlation of 12 continuous numerical input variables was analyzed using Spearman correlation test, as input variables are not normally distributed. In the correlation heatmap (Fig. 3), at least seven pairs of features (such as UI-Z and Sn-Z, Overjet and ANB) showed highly correlations (either positive or negative). The multicollinearity of input features could lead to bias on regression coefficients. To decline the impact of multicollinearity on prediction results, the data were pretrained with a ridge regression algorithm (training details can be reached in Supplementary Table 2), and obtained weighting coefficients were assigned to input variables.

### Modeling and network architecture

**Fig. 4:**
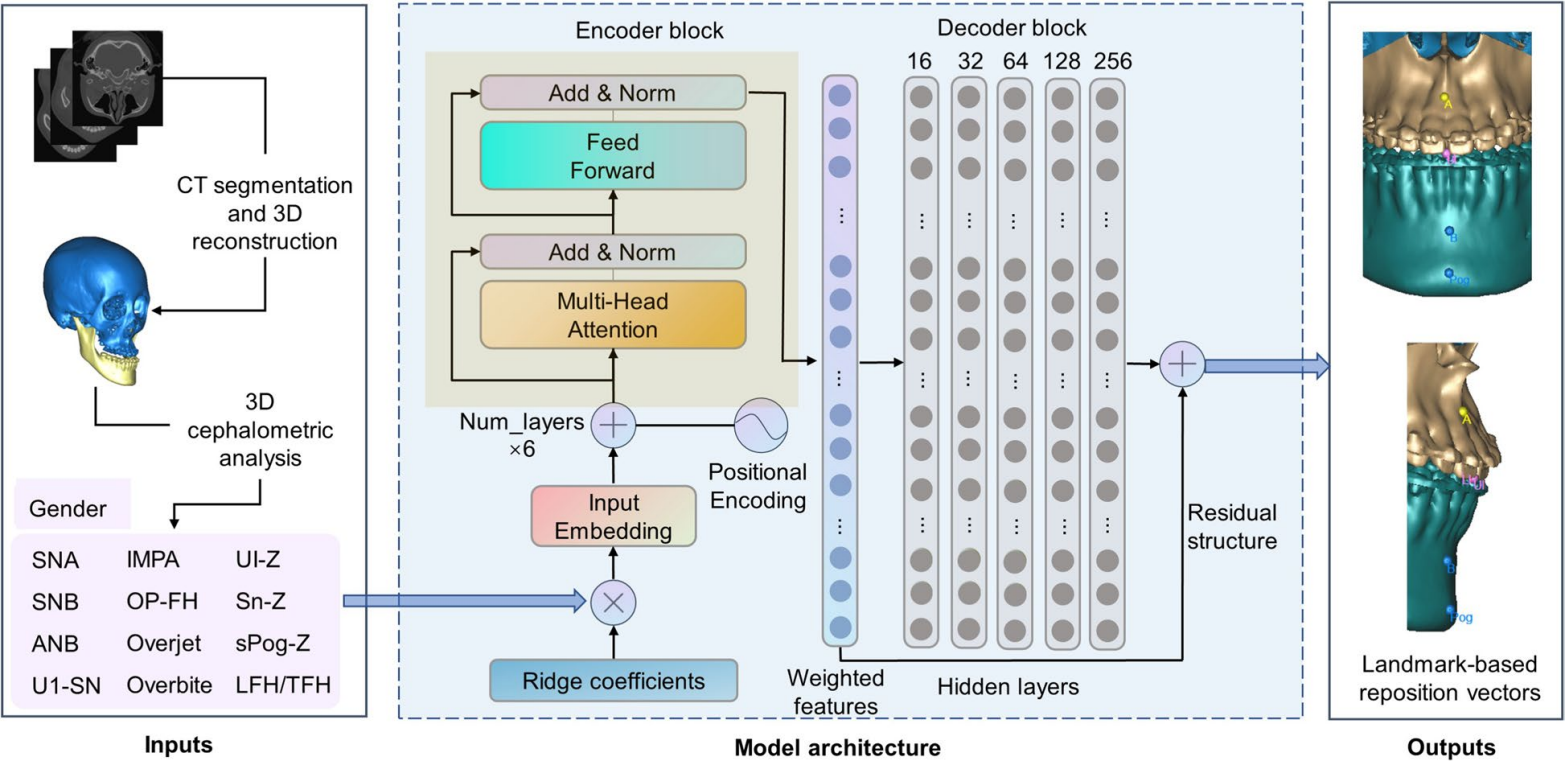
Architecture of VSP transformer. Norm: normalization. CT-reconstructed CMF model was quantified using 3D cephalometric analysis. 12 cephalometric variables and gender were weighted using ridge coefficients. After embedding, the data were encoded with positional information and then input into encoder block to extract features. At last, the extracted features were decoded by a MLP and an embedded residual structure to obtain output variables

The architecture of our proposed model, VSP transformer, is shown in Fig. 4. Since 10 outputs were continuous numerical variables, a regression model was required to establish the mapping relationship between input and output variables. Transformer [11] was employed as the backbone of the model framework. Using sine and cosine functions of different frequencies, input data were encoded with positional information to avoid missing features. The data were then input into encoder block, which was a stack of 6 encoders. Each encoder consisted of 2 multi-head attentions, 2 trainable layer normalization and a fully connected feed forward network. Meanwhile, 2 sub-layers of one encoder was connected by a residual structure to guide information and gradient flow. The decoder block was a multi-layer perceptron (MLP), which had 5 hidden layers. The shape of neurons was set as [16,32,64,128,256]. Relu was chosen as the activation function. A residual structure was added between the input and output layer of the decoder block, so that complexity of the model can be adjusted dynamically to prevent degradation.

### Training details

In total, we trained five classical regression models for the prediction. Architectures and training details of the models other than VSP transformer can be reached in Supplementary Table 2. Three metrics, coefficient of determination value (R^2^ score), mean square error (MSE) and mean absolute error (MAE), were employed to quantify their prediction performance. They were calculated using the following equations:

2 $${R}^{2}=1-\frac{{{\sum }_{i=1}^{n}({y}_{i}-\stackrel{\wedge }{{y}_{i}}{)}^{2}}}{{\sum }_{i=1}^{n}({y}_{i}-\stackrel{\_}{{y}_{i}}{)}^{2}}$$

3 $$MSE=\frac{1}{n}{\sum }_{i=1}^{n}({y}_{i}-\stackrel{\wedge }{{y}_{i}}{)}^{2}$$

4 $$MAE=\frac{1}{n}{\sum }_{i=1}^{n}{\left|{y}_{i}-\stackrel{\wedge }{{y}_{i}}\right|}$$

where *n* represents the total sample, ${y}_{i}$ represents the sample label,$\stackrel{\wedge }{y}$ represents the predicted value of the sample, and,$\stackrel{\_}{y}$ represents the average value of the sample.

VSP transformer was built using Pytorch deep learning framework. From the development dataset of 383 patients, 90% samples were randomly selected as the training set of the model (*n* = 345). The remaining samples were used as an internal validation set (*n* = 38) to evaluate the fitting effect. Samples in training set were divided into ten equal batches randomly. The network was optimized by individual backpropagation process and gradient descending with gradients estimated by Adam optimizer. MAE was selected as loss function of each epoch, during which the learning rate gradient of different parameters was estimated. The epoch was 600. The batch size was 34. The learning rate was 1e-3 and the decaying rate was 1e-4.The dropout was 0.1.

### Contribution calculation of input features

To reveal the decision-making process of the model and illustrate more interpretable results for clinicians, permutation importance (PI) method [12] was employed to obtain the contribution ranking of 13 input features. In calculation process, 13 columns of features were shuffled 500 times. MAE was set as the loss function to calculate changes before and after shuffling because MAE is sensitive with prediction error variation. Compared with other methods for measurement of the feature importance, PI demonstrates low computational cost, and the process of random shuffling does not change the parameters inside the model.

## Results

### Baseline characteristics of patients

The mean age of patients was 23.3 ± 4.5 years in development dataset and 25.0 ± 4.7 years in clinical test dataset. The population was predominantly female in both datasets. The distribution of skeletal classification of deformity and performed surgery type were balanced in two datasets. The datasets derived from four chief surgeons to reduce inter-surgeon bias. All baseline characteristics of patients were summarized in Table 1.

**Table 1:** Baseline characteristics of patients in the dataset

Characteristics	Development set (No. = 383)	Clinical test set (No. = 49)
Gender—No. (%)		
Male	140(36.6)	10(20.4)
Female	243(63.4)	39(79.6)
Age (in year)	23.3 ± 4.5	25.0 ± 4.7
Skeletal Classification—No. (%)		
I	83(21.7)	10(20.4)
II	66(17.2)	8 (16.3)
III	234(61.1)	31(63.3)
Surgery Type^*^- No. (%)		
L + B	297(77.6)	31(63.3)
L + B + G	61(15.9)	11(22.4)
L	1(0.3)	0(0)
L + G	0(0)	0(0)
B	17(4.4)	4(8.2)
B + G	7(1.8)	3(6.1)
Chief Surgeon—No. (%)		
Yu	184(48.0)	22(44.9)
Shen	76(19.9)	10(20.4)
Shi	64(16.7)	11(22.4)
Zhang	59(15.4)	6(12.3)

^*^ For surgery type, L is short for Le Fort I; B is short for BSSRO; G is short for Genioplasty

### Comparison with other regression models

The best performances of five models were listed in Table 2. The prediction accuracy of ridge regression and VSP transformer was almost equally high in validation set. While in clinical test set, R^2^ score decreased much more in ridge regression than VSP transformer, which suggested VSP transformer possessed the best generalization ability. Considering its robustness and high accuracy, VSP transformer model was determined as the most suitable model for the prediction task and to take following examinations.

**Table 2:** Model performance on prediction

Models	Validation set			Clinical test set		
	**R**^**2**^** score**	**MSE**	**MAE(mm)**	**R2 score**	**MSE**	**MAE(mm)**
Random Forest	0.4859	0.0138	1.7322	0.4564	0.0083	1.4088
KNN	0.4599	0.0154	1.9200	0.4337	0.0088	1.4978
Ridge Regression	0.6592	0.0094	1.3568	0.4390	0.0084	1.3741
ANN	0.5467	0.0144	1.6722	0.4914	0.0080	1.3614
VSP transformer	0.6602	0.0078	1.4137	0.5159	0.0075	1.3440

MSE were calculated by normalization results. MAE were calculated by de-normalization results

### Prediction performance of VSP transformer

**Fig. 5:**
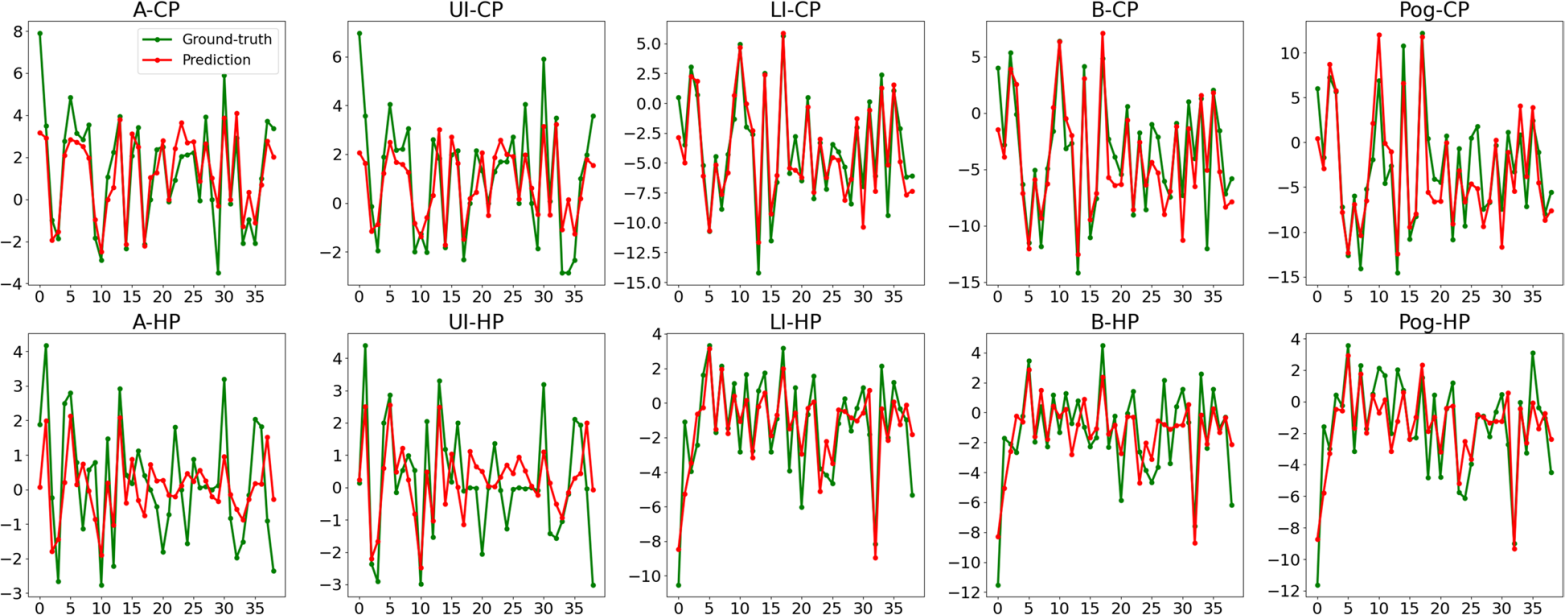
Prediction errors of ten output variables in validation set. Green dots represent ground truth and red dots represent predicted values

**Fig. 6:**
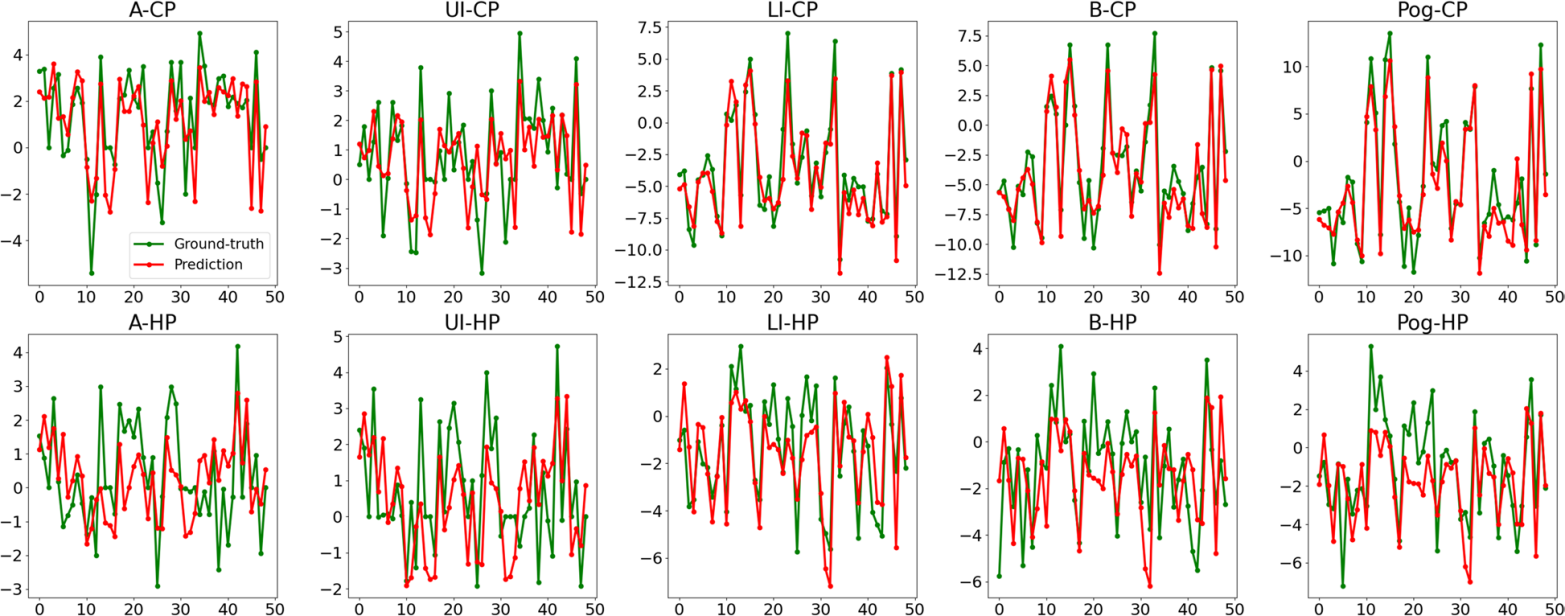
Prediction errors of ten output variables in clinical test set. Green dots represent ground truth and red dots represent predicted values

To evaluate prediction performance of VSP transformer in detail, R^2^ score and MAE were also calculated for each output variable separately (Table 3). The predicted values and ground truth of each output variable were plotted in Fig. 5 (validation set) and Fig. 6 (clinical test set). Based on the results, variables represented sagittal movement (x-CP) had higher R^2^ score than variables represented vertical movement (x-HP), suggesting that the model was able to predict more accurate results of sagittal movement than vertical movement. Six of ten MAEs of clinical test set were smaller than according MAEs in validation set, which is to some extent consistent with prediction results of other four models in Table 1––despite R^2^ score decreased in test set, MAE in test set was still smaller than validation set. This consequence might partially result from the range difference of output variables between validation and test datasets.

**Table 3:** Model prediction accuracy of each output variable

Output variables	Validation set		Clinical test set	
	**R2 Score**	**MAE(mm)**	**R2 Score**	**MAE(mm)**
A-CP	0.7274	1.0312	0.4645	1.2530
A-HP	0.3354	1.1822	0.1858	1.1594
UI-CP	0.7778	1.9016	0.4424	1.0810
UI-HP	0.6354	1.5137	0.2256	1.2071
LI-CP	0.8589	1.3247	0.8759	1.1614
LI-HP	0.7176	1.2836	0.6118	1.1582
B-CP	0.7801	2.4293	0.8558	1.4263
B-HP	0.7023	1.2707	0.1924	1.6661
Pog-CP	0.6123	1.1507	0.8911	1.7965
Pog-HP	0.4542	1.0494	0.4147	1.5325

To further characterize the performance of this model, we examined how frequently the models' prediction fell within a given error margin in clinical test set (Table 4). Ten output variables were divided into four groups according to regions (maxilla or mandible) and dimensions (sagittal or vertical) they belonged to. Prediction accuracy was calculated based on groups. Notably, maxillary reposition vectors showed higher accuracy than mandibular reposition vectors. The prediction accuracy of maxilla was almost equally high for sagittal and vertical reposition vectors, while in terms of mandible, the prediction accuracy for vertical reposition vectors was much lower than sagittal reposition vectors.

**Table 4:** Model prediction accuracy within a given margin in different regions and dimensions (clinical test set)

Variables	Error margin	Model accuracy(%)
Maxi-sagittal	± 1 mm	51.02
	± 2 mm	93.88
	± 3 mm	100
Maxi-vertical	± 1 mm	55.10
	± 2 mm	89.79
	± 3 mm	100
Mandi-sagittal	± 1 mm	51.02
	± 2 mm	85.71
	± 3 mm	93.88
Mandi-vertical	± 1 mm	32.65
	± 2 mm	69.39
	± 3 mm	97.96
All outputs	± 1 mm	50.41
	± 2 mm	81.84
	± 3 mm	94.89

Maxi-sagittal includes A-CP and UI-CP. Maxi-vertical includes A-HP and UI-HP. Mandi-sagittal includes LI-CP, B-CP and Pog-CP. Mandi-vertical includes LI-HP, B-HP and Pog-HP

### Contribution of each input feature

**Fig. 7:**
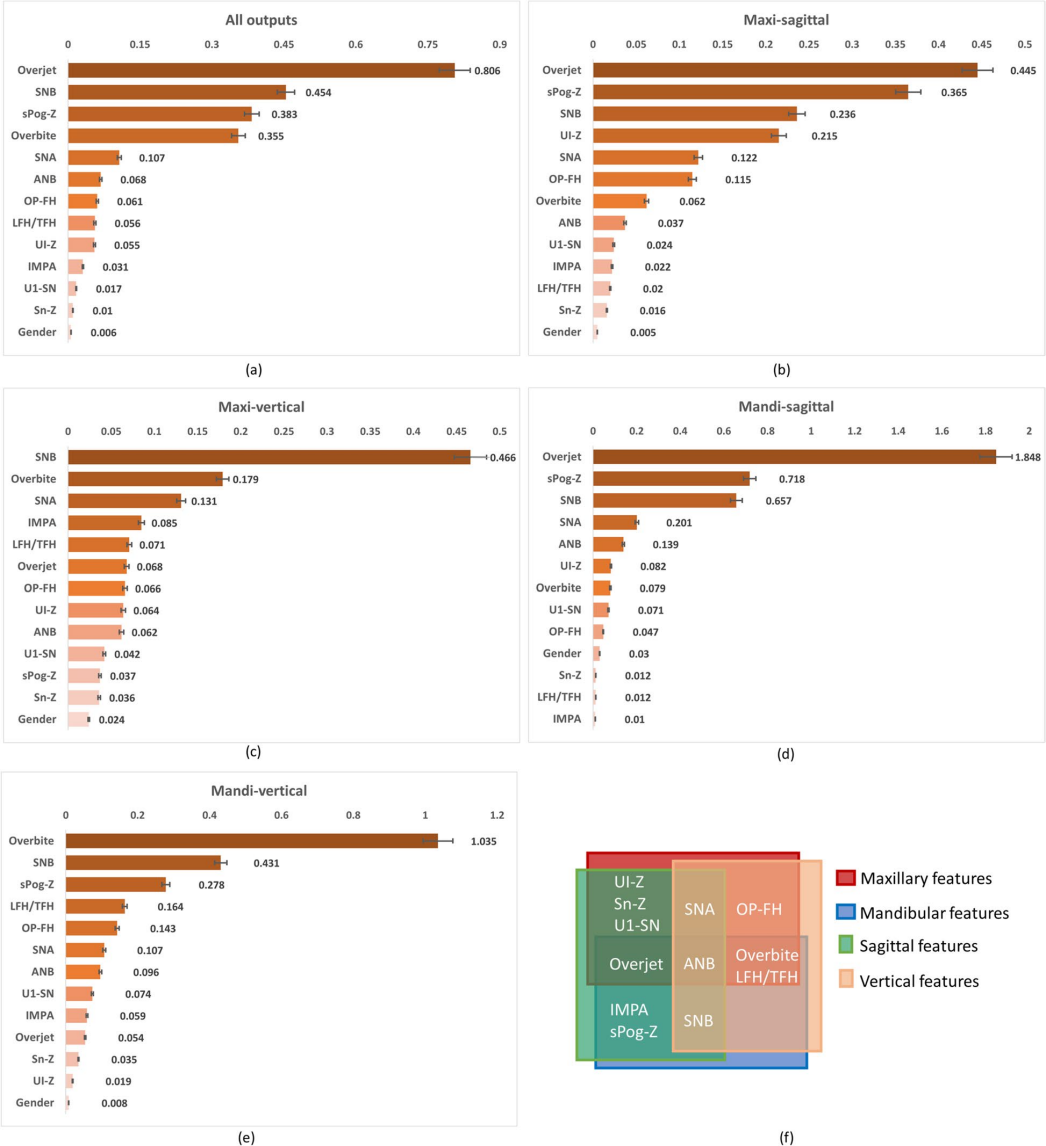
**a-e** Contributions of input feature. **f** properties of 12 cephalometric features

The feature contribution analysis was conducted based on all outputs and four subcategories as well (Fig. 7). Higher score represents higher contribution for final decision. The ranking results showed a lot of consistency with clinical knowledge and experience. Overjet, SNB, sPog-Z and Overbite were the most important features and Gender was the least important feature (Fig. 7a). 12 cephalometric features demonstrated different rank order in four subcategories of all outputs (Fig. 7b-e). For clearly presentation, 12 cephalometric features were divided into different regions in Fig. 7f based on their properties. Purely vertical features (OP-FH, Overbite and LFH/TFH) had higher ranks in maxi-vertical (i.e. A-HP and UI-HP) and mandi-vertical (i.e. LI-HP, B-HP and Pog-HP) groups (Fig. 7c,e). Other than maxillary features (UI-Z, SNA and overjet), purely mandibular features (sPog-Z and SNB) also had high ranks in Maxi-sagittal (i.e. A-CP and UI-CP) group (Fig. 7b). Features of facial soft tissue (UI-Z, Sn-Z and sPog-Z) made fairly contributions for prediction, especially sPog-Z.

## Discussion

This study proposed a deep learning method for prediction of orthognathic surgery planning. Comparing with other researches [4, 6–8], we achieved higher prediction accuracy and greater practical-effectiveness. In [4, 6, 7], they adopted the strategy of predicting normal bone shape through deformation network to assist VSP. Despite of their impressive outcome, shape of dentition and tooth was concomitantly deformed. As a result, the predicted normal bone model can neither meet the high accuracy requirement of rebuilding occlusion nor be used for manufacturing surgical guides. Ma et al. (2022) proposed a different strategy for predicting orthognathic surgical plan [8]. The cascade neural network they developed was able to predict postoperative 3-dimesional coordinates of 11 specific landmarks. The landmarking results were transformed into vectors of jawbones' movement. Based on the data, predicted post-operative model was visualized through manually repositioning pre-operative bony segments. This method was tested on 6 real patients' data, and the average prediction accuracy was 5.4 ± 0.6 mm at the landmark level. In our study, we adopted a similar strategy, in which reposition vectors of bony segments instead of normal bone shape were predicted. In clinical practice, orthognathic surgery was usually performed by repositioning bony segments without changing its own morphology (only relative positional relationships among maxilla, mandible and cranium were changed). Hence, surgical planning guided by reposition vectors (numerical variables) will generate more realistic and practical outcomes for patients. Meanwhile, to increase prediction accuracy, CMF structures (bones, occlusion and facial soft tissue) were quantified using 3D cephalometric analysis—a widely used knowledge system to assist diagnosis and treatment planning. Eventually, Prediction errors at landmark level in this study (1.34 mm) were significantly lower than in the study of Ma et al. (5.4 ± 0.6 mm) [8]. Although only five landmarks were selected to calculate reposition vectors, it’s enough to determine the most elaborate vectors of maxilla-mandible complex, namely translation along X axis, translation along Z axis and Pitch [2]. The remaining three reposition vectors, namely translation along Y axis, Yaw, Roll were relatively easy to determine based on symmetry principle in VSP. Therefore, transverse movement was not included in output variables. In addition, it's worth noting that different surgeons could have remarkably different designs for a specific case. Therefore, our datasets were collected from four chief surgeons to reduce potential experience dependence or biases. Overall, our approach for prediction is closer to surgeon's decision-making pattern and showed good prediction accuracy.

Another important contribution of this research is that we analyzed correlations between input features. Except for gender, input features were continuous numerical variables. Correlation of input features was observed based on Spearman correlation coefficients. The data demonstrated severe multicollinearity—at least seven pairs of highly correlated features. According to current knowledge, some of the correlations weren't expected, such as U1-SN and SNB (r = 0.68). Larger dataset can be analyzed in future to detect if some constant patterns of dentofacial deformities exist.

For an AI-based prediction model in medicine, clinicians expect more than to transfer valuable decision-making process of experienced surgeons to the model. The ability to present larger amounts of interpretable information is of great importance to augment surgeons' clinical judgements and gain their trust [13]. When the diagnosis or treatment planning are inconsistent among different doctors, it is important that an AI-based model can provide valuable information to assist decision and decrease biases [14]. Therefore, an interpretable model with suboptimal decisions is better than a highly accurate model without any interpretable information [15]. Unfortunately, correlations of the features will greatly decrease the model interpretability. To enhance interpretability and reduce possible bias resulted from data multicollinearity, Transformer architecture was employed for this non-linear relationship prediction and PI method was introduced for the model to reveal features' contribution. Transformer was first proposed for the machine translation [11] and achieved state-of-the-art performance in various tasks in nature language processing (NLP) field. Multi-head attention mechanism of Transformer can adjust attention distribution flexibly and align the token relationship between two sequences when processing the global information [16], so self-attention can enhance interpretability of DL-based model. Meanwhile, PI method was employed to reveal the decision-making process of VSP transformer without interrupting original model parameters, and the results showed a lot of consistency with clinical knowledge and experience (Fig. 7).

To further accomplish end-to-end prediction in Fig. 1, there are still some researches to do. First of all, surgery types and reposition vectors should be predicted simultaneously to complete a whole surgical plan. Facial midline also needs to be detected automatically to evaluate symmetry and determine transverse reposition vectors [17]. Occlusion prediction can be more concrete and accurate by utilizing digital final occlusion model as a reference. Moreover, surgeons need to communicate predicted outcomes (mainly about facial appearance) to the patient and adjust surgical plan according to patient’s own anticipation and personality. Therefore, accurate prediction of postoperative facial changes is also of great importance to complete a personized and optimal surgical design [18, 19]. At last, larger and multicenter real-patient dataset needs to be collected since big data accumulation of cephalometric results will bring better understanding of facial deformity and aesthetics, therefore reduce bias in decision.

## Conclusions

In summary, a transformer-based regression neural network named VSP transformer was proposed to predict orthognathic surgery plan. Evidence conformed that features of facial soft tissue were important for surgical planning. VSP transformer demonstrated enhanced interpretability and good generalization despite of data multicollinearity. The model can be regarded as a promising alternative to assist VSP for orthognathic surgery.

## Supplementary Information

**Additional file 1**: **Supplementary Table 1.** Definitions of 3D skeletal, dentoalveolar and soft tissue cephalometric variables. **Supplementary Table 2.** The parameters of the four baseline models.

## Data Availability

The datasets generated and/or analyzed during the study are not publicly available currently because the Institutional Review Board of Shanghai Ninth People’s Hospital, College of Stomatology, Shanghai Jiao Tong University School of Medicine did not allow it. However, the data are available from the corresponding author on reasonable request.

## Abbreviations

3D: 3-Dimensional
VSP: Virtual surgical planning
CMF: Craniomaxillofacial
PI: Permutation importance
TMJ: Temporomandibular joint
AI: Artificial intelligence
DL: Deep learning
CT: Computed tomography
BSSRO: Bilateral sagittal split ramus osteotomy
FH: Frankfurt horizontal plane
HP: Horizontal plane
SP: Middle sagittal plane
CP: Coronal plane
NHP: Natural head position
A: A point
UI: Midpoint of U1 incisal tip
LI: Midpoint of L1 incisal tip
B: B point
Pog: Pogonion point
MLP: Multi-layer perceptron
MAE: Mean absolute error
R2 score: Coefficient of determination value
MSE: Mean square error
NLP: Nature language processing
