# Supplementary material for: Prediction of orthognathic surgery plan from 3D cephalometric analysis via deep learning
Source: BMC Oral Health. 2023 Mar 18;23:161. doi: 10.1186/s12903-023-02844-z (PMC10024836; doi:10.1186/s12903-023-02844-z)
Supplement: Supplementary file 1 — Additional file 1: Supplementary Table 1. Definitions of 3D skeletal, dentoalveolar and soft tissue cephalometric variables. Supplementary Table 2. The parameters of the four baseline models. [file 12903_2023_2844_MOESM1_ESM.docx]

**Prediction of orthognathic surgery plan from 3D cephalometric analysis via deep learning**

**Journal name:** BMC Oral Health

**Author information:**

Mengjia Cheng ^a, b, c.1^, Xu Zhang ^d.1^, Jun Wang ^e^, Yang Yang ^f, g^, Meng Li ^a, b, c^, Hanjiang Zhao ^a, b, c^, Jingyang Huang ^a, b, c^, Chenglong Zhang ^a, b, c^, Dahong Qian ^g.*^, Hongbo Yu ^a, b, c.*^

a. Department of Oral and Cranio-maxillofacial Surgery, Shanghai Ninth People’s Hospital, College of Stomatology, Shanghai Jiao Tong University School of Medicine, Shanghai 200011, China;

b. National Clinical Research Center for Oral Diseases, Shanghai 200011, China;

c. Shanghai Key Laboratory of Stomatology & Shanghai Research Institute of Stomatology, Shanghai 200011, China.

d. Mechanical college, Shanghai Dianji University. Shanghai 201306, China

e. School of Computer & Computing Science, Zhejiang University City College, Hangzhou 310000, China.

f. Shanghai Lanhui Medical Technology Co., Ltd, Shanghai 200333, China

g. School of Biomedical Engineering, Shanghai Jiao Tong University, Shanghai 200030, China.

^1^ Mengjia Cheng and Xu Zhang have contributed equally to this work.

^*^ Corresponding author: Email address: [yhb3508@163.com](mailto:yhb3508@163.com%20(Yu) (Hongbo Yu), Dahong.qian[@sjtu.](mailto:yhb3508@163.com%20(Yu)edu.cn (Dahong Qian)

1. **Definitions of cephalometric variables**

| **Supplementary Table 1. Definitions of 3D skeletal, dentoalveolar and soft tissue cephalometric variables.** | |
| --- | --- |
| **Variables** | **Definition[1, 2]** |
| SNA | The angle between landmarks S, N, and A |
| SNB | The angle between landmarks S, N, and B |
| ANB | The angle between landmarks A, N, and B |
| UI-SN | The angle between long axis of upper incisors and a plane passing through S, N and normal to SP. |
| IMPA | The angle between long axis of lower incisors and mandibular plane(MP) |
| OP-FH | the angle between maxillary occlusal plane (OP) and FH |
| Overjet | Sagittal distance between UI and LI point |
| Overbite | Vertical distance between UI and LI point |
| UI-Z | Sagittal distance between UI and zero-degree meridian |
| Sn-Z | Sagittal distance between Sn (Point Subnasale) and zero-degree meridian |
| sPog-Z | Sagittal distance between sPog (the most anterior midpoint of the chin soft tissue) and zero-degree meridian |
| LFH/TFH | The ratio of lower facial height(ANS-Me) and total facial height(N-Me) |

1. **Architectures and training details of four baseline models**
   1. Ridge Regression Model

Ridge regression is a tool to analyze the data suffered by multicollinearity. It is a phenomenon when two or more variables in multiple regression are highly linear related. If there is multicollinearity in the data, least squares estimation is unbiased; it is in contrast if the over fitting occurs[3]. Ridge regression model is similar to least squares estimation; unless Ridge coefficient variable is added and optimized. It is expected that the net effect will provide more reliable estimation. The Ridge coefficients are used to reduce a penalized residual sum of squares.

- 1. Random Forest regression (RFR)

RFR belongs to the Bagging algorithm, and Bagging is a method of ensemble learning (the ensemble learning method is roughly divided into Boosting and Bagging methods)[4]. The general idea of ensemble learning is to train multiple weak models and package them together. A strong model is formed, and the performance of the strong model is much better than that of a single weak model (three cobblers top one Zhuge Liang. Note: the weak and strong here are relative), and the weak model can be a decision tree, SVM and other models, In random forests, decision trees are used for weak models.

In the training phase, random forest uses bootstrap sampling to collect multiple different sub-training datasets from the input training dataset to train multiple different decision trees in turn; in the prediction phase, random forest averages the prediction results of multiple internal decision trees to obtain final result.

- 1. K-nearest neighbors(KNN)

KNN algorithm is a very special machine learning algorithm because it has no learning process in the general sense. Its working principle is to use the training data to partition the feature vector space, and the partition result as the final algorithm model. There is a sample data set, also known as the training sample set, and the existence of each data sample concentration, no label input data, after the no label data of each characteristic compared with data corresponding to the characteristics of sample set, and then the most similar sample feature extracting data (nearest neighbor).Finally, find the K-nearest neighbors of the new prediction instance, and then de-mean the target value of these K samples to be used as the prediction value of the new sample. Generally speaking, we only select the first K most similar data in the sample data set, which is the origin of K in KNN algorithm.

- 1. Artificial Neural network(ANN)

ANN emulates function of human brain. Moreover, it simplifies and derives a structure of biological neurons. Generally, neural network model comprises input, hidden and output layers .Nodes in the layers imitate biological neurons; and the nodes are connected by applying different weights. Algorithms of neural network includes input data forward propagation and error back propagation. The original data in forward propagation is transferred from input to hidden layer. Then, the hidden layer extracts some data features by applying activation function. Activation function aims to generate a non-linear decision boundary by performing non-linear combinations of weighted inputs.

| **Supplementary Table 2.** **The parameters of the four baseline models** | |
| --- | --- |
| **Model** | **Parameters** |
| Ridge Regression Model | alpha=0.1, fit_intercept=False, normalize=True, copy_X=True, max_iter=3000, tol=1e-4, solver='auto' |
| Random Forest regression | n_estimators = 631, min_samples_split = 5, min_samples_leaf= 4, max_features='auto', max_depth =8, random_state=0 |
| K-nearest neighbors | n_neighbors=13, weights='uniform', algorithm='auto', leaf_size=100, p=1, metric_params=None,n_jobs=-1 |
| Artificial Neural Network | No. of layer=2, No. of neuron in a hidden layer=[16,32,64,128,256] |

**References**

1. Farronato M, Baselli G, Baldini B, Favia G, Tartaglia G M. 3D Cephalometric Normality Range: Auto Contractive Maps (ACM) Analysis in Selected Caucasian Skeletal Class I Age Groups. Bioengineering-Basel. 2022; 9(5) , doi:10.3390/bioengineering9050216

2. Wang R H, Ho C T, Lin H H, Lo L J. Three-dimensional cephalometry for orthognathic planning: Normative data and analyses. J Formos Med Assoc. 2020; 119(1 Pt 2):191-203.

3. Leauprasert K, Suwanasri T, Suwanasri C, Poonnoy N. Intelligent Machine Learning Techniques for Condition Assessment of Power Transformers. 2020; in 2020 International Conference on Power, Energy and Innovations (ICPEI).

4. Shanmugasundar G, Vanitha M, Čep R, Kumar V, Kalita K, Ramachandran M. A Comparative Study of Linear, Random Forest and AdaBoost Regressions for Modeling Non-Traditional Machining. Processes. 2021; 9(11), doi: 10.3390/pr9112015
